# Magnetic Resonance Conditional Microinjector

**DOI:** 10.3390/jimaging5010004

**Published:** 2018-12-30

**Authors:** Adam Wineland, Yue Chen, Brian Boland, Kevin Chan, Zion Tse

**Affiliations:** 1 School of Electrical and Computer Engineering, University of Georgia, Athens, GA 30602, USA; 2 Department of Mechanical Engineering, University of Arkansas, Fayetteville, AR 72701, USA; 3 Neuroimaging and Visual Science Laboratory, Departments of Ophthalmology and Radiology, NYU School of Medicine, New York University, New York, NY 10016, USA

**Keywords:** glaucoma, intraocular pressure, blindness, microinjector, eye, MRI, image guided, actuation, pneumatic, magnetic resonance

## Abstract

Glaucoma, one of the leading causes of blindness, has been linked to increases in intraocular pressure. In order to observe and study this effect, proposed is a specialized microinjector and driver that can be used to inject small amounts of liquid into a target volume. Magnetic resonance imaging (MRI) guided remotely activated devices require specialized equipment that is compatible with the MR environment. This paper presents an MR Conditional microinjector system with a pressure sensor for investigating the effects of intraocular pressure (IOP) in near-real-time. The system uses pressurized air and a linear actuation device to push a syringe in a controlled, stepwise manner. The feasibility and utility of the proposed investigative medical research tool were tested and validated by measuring the pressure inside an intact animal donor eyeball while precise, small volumes of water were injected into the specimen. Observable increases in the volume of the specimen at measured, specific target pressure increases show that the system is technically feasible for studying IOP effects, while the changes in shape were depicted in MRI scan images themselves. In addition, it was verified that the presence and operation of the system did not interfere with the MRI machine, confirming its conditional compatibility with the 3T MRI.

## 1. Introduction

Glaucoma is the second leading cause of permanent blindness, affecting over 2.7 million adults over the age of 40 in the United States alone as of 2009. The two most common types of glaucoma are called ‘open-angle’ and ‘angle-closure.’ Both kinds of glaucoma can be caused by high pressure inside the eye. Open-angle glaucoma is a condition that develops slowly over time and is characterized by a long-term clogging of the inside of intraocular drainage canals. Angle-closure glaucoma can develop quickly and is caused by a blockage at the entrance of the drainage canals [1]. Over time, excessive intraocular pressure (IOP) is considered to be a major risk factor for blindness by damaging the retinal ganglion cell (RGC) axonal function. These axons are responsible for passing information through the optic nerve head (ONH) to the brain. Force from excessive IOP can displace the sclera and put pressure on the ONH. This has been an area of interest to researchers who have developed experiments designed to study the biomechanics of the sclera to better determine the phases of this process and possible causes. A thorough understanding of this process is necessary prior to developing interventional procedures [2,3].

**Figure 1:**
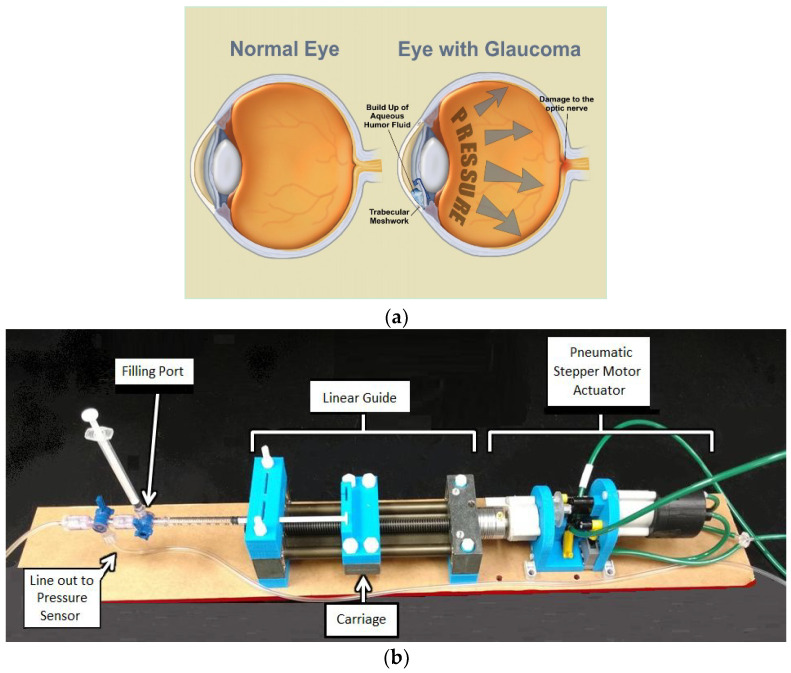
(**a**) The effects of glaucoma illustrated [11]. (**b**) A broadly labeled image of the microinjector system setup with syringe.

For the purpose of studying tissue deformation, several methods have been developed, including: uniaxial extension, biaxial extension, and inflation testing [4,5,6]. Uniaxial and biaxial tests involve excising strips of tissue and stretching the tissue using controlled stresses. Inflation tests, however, involve measuring deformations of the corneoscleral shell while simultaneously inflating the donor eyeball in vitro using controlled pressurization. Inflation tests are beneficial when compared to those methods because they can be performed on excised posterior scleral shells or on whole globes. Furthermore, compared to excising strips of tissue, inflation testing on a whole globe better approximates the normal biomechanical state of the eye [5] (Figure 1a). After discussing inflation testing, a common method of performing such testing, microinjection, will be explored.

The aim of the research conducted was to develop a precise microinjector system capable of operating within the constraints of a magnetic resonance imaging (MRI) environment, so that deformations in ocular tissue may be observed using MRI at a larger scale than by using a microscope. This was done to develop a proof of concept for a research tool that could be used to explore intraocular pressure and how it could cause glaucoma and lead to blindness. As the pressure is increased in small increments, verified by a sensitive pressure gauge, the overall shape or general volume of the specimen can be determined at each increment. This can show what ranges of pressure are necessary to visibly affect the appearance of the eye. Furthermore, ideally the tests would also indicate which areas of the eye are most vulnerable to the pressure increases, possibly indicating a target area for anti-inflammatory drug treatments or for surgical interventions. Currently, there is no procedure that has been proven to eliminate IOP and prevent or cure glaucoma. The hope is that with enough information, doctors could hypothesize a procedure for relieving IOP in high risk patients.

We also set out to demonstrate, as a proof of concept, how our microinjector system is made up of mostly 3D printed parts, can incrementally increase IOP and gather pressure sensor control feedback, and that it has minimal effect on the signal to noise ratio (SNR) of the MRI scans taken of the animal donor eye specimen.

Typically, the equipment required for inflation testing includes a fluid injection system, a container to hold the specimen, a humidity chamber, a device to measure displacement, and a device to measure thickness [5]. Elsheikh et al. studied the modulus of elasticity of the human cornea by using a custom pressure chamber to inflate excised corneas. Changes in tissue were measured with charge-coupled device (CCD) cameras and a laser measurement system. Coudrillier et al. used a syringe injection system to inflate excised posterior scleral shells and CCD cameras to monitor tissue changes [2,3,4,5,6]. Ho et al. used a gravity perfusion system to inflate whole globes and magnetic resonance imaging (MRI) to study the effects of excessive IOP [4]. The average pressure in the human eye is relatively low (about 12 mmHg to 22 mmHg) [3] and pressure needs to be increased in small steps during experiments. The inflation systems used by Elsheikh et al. and Coudrillier et al. can inflate an eye in small increments, but are not compatible with magnetic resonance (MR) environments. The gravity perfusion system is acceptable for use in MR environments, but is incapable of executing precise, controlled increments. Therefore, researchers are in need of a device capable of injecting liquid in small amounts while in the vicinity of an MRI scanner [6]. This paper focuses solely on the first piece of equipment required for inflation testing: a fluid injection system. Specifically, the aim of the study was to develop a programmable, MR usable pump capable of inflicting small, precise, incremental increases in the pressure inside a specimen by injecting water.

Conventional microinjection involves using glass micropipettes with diameters between 0.5 µm to 5 µm to inject small volumes of liquid, such as drugs or macromolecules, into a target cell or other microscopic target [7]. One micromanipulator is used to position the target, and the other is used to maneuver the micropipette [8]. In contrast, this study aims to develop the equipment needed to perform macro-level analysis of tissue deformation under MRI by injecting liquid into small areas of an intact, harvested specimen. The additional information provided at this scale can help to better understand the effects of intraocular pressure.

## 2. Materials and Methods

For the injections, pulsed flow systems with precise increments are preferable but usually are more expensive [9]. To solve the problem of achieving measurable effects under MR constraints, Harvard Apparatus (Holliston, MA, USA) and Chemyx (Stafford, TX, USA) each offer an MR Conditional syringe pump, but their limitations include minimum safe distances from the MRI bore and certain anchoring requirements, respectively [10]. Those imposed restrictions in addition to the expensiveness of such systems make their use cost-prohibitive for many researchers.

Our device adheres to MR constraints with a short 1 m tube and is constructed of non-ferromagnetic materials. A small, pneumatic stepper motor is attached to one end of a linear guide (SWX-104001, Igus, East Providence, RI, USA). A syringe is attached to the other end of the linear guide using customized adaptors. At the end of the syringe is an apparatus for filling the tubing with water. The apparatus is also used for removing air bubbles from the system and equalizing the pressure in the tubing after the setup process. Figure 1b shows the device in its current configuration.

The control system uses a laptop running a computer program called LabVIEW, a DAQ card (USB-6009, National Instruments, Austin, TX, USA), two three-way pneumatic solenoid valves (3AK-1/8-G, WIC Valve, Silicon Valley, CA, USA), a valve driver circuit to power the valves (Compact L298 Motor Driver, Solarbotics Ltd., Calgary, AB, Canada), a pressure sensor (SSCDANT005PGAA5, Honeywell, Golden Valley, MN, USA), and a pressurized source of pressurized air.

The working principle is that the shaft of the mostly three dimensional (3D) printed pneumatic stepper motor, which has been developed, tested, and published previously, rotates a lead screw causing the carriage to move forward or backward based on the rotation direction [12,13]. Our unique motor and injector combination is what we believe to be the first to incorporate closed-loop feedback from a pressure sensor. One key factor for adopting the stepper motor design specifically is its deterministic nature under typical operating conditions. Because the plunger position is set based on number of steps, the inherent volume is also based on that number. While caused by the aforementioned volumetric fluid displacements, they were measured by the system in real-time using highly accurate industrial grade sensors (discussed in detail in Table 1). No system calibration is required because of this.

For each full rotation of the lead screw, the carriage will advance 2 mm. As this stepper motor advances 3.6 degrees per step, the carriage moves 0.02 mm per step. The volume of fluid injected depends on the size of the syringe. The injector was designed to hold three different sizes of syringes (Nipro Medical Corporation, Bridgewater, NJ, USA). The largest syringe for the microinjector holds 10 mL per 61 mm, or approximately 0.164 mL/mm. Therefore, for each step of the motor, the liquid inside the syringe is displaced by about 0.00328 mL, or 3.28 µL. The medium-size syringe holds 3 mL per 42 mm, which is approximately 0.0714 mL/mm. For each step, the liquid is displaced by approximately 1.43 µL. The smallest syringe holds 1 mL per 57.25 mm, or approximately 0.0175 mL/mm. Therefore, liquid is displaced by approximately 0.349 µL per step. Table 1 compares parameters of the three different syringe sizes.

During the trial for each condition tested, the operator may set a desired volume to be injected or set a desired applied pressure. For the first design case, after the specified volume has been displaced, the program is written to go to standby, and the stepper motor idles. Likewise, for the latter case, after a desired applied pressure is achieved, the device stops pumping and resorts to standby.

Testing the efficacy of the microinjector involved inserting a non-magnetic catheter needle (NIC-20GX2”, Nipro Medical Corporation, Bridgewater, NJ, USA) into a series of sheep eyeballs (Carolina Biological Supply Company, Burlington, NC, USA) and injecting water into an eyeball in order to inflate and stretch the sclera. Initial tests involved capturing images of an eyeball using an 8 megapixel camera set to a time-lapse mode. MRI was later used to view an eyeball’s interior anatomical structure. For experiments utilizing the camera, the pressure was logged every five seconds, and images were captured every thirty seconds.

**Figure 2:**
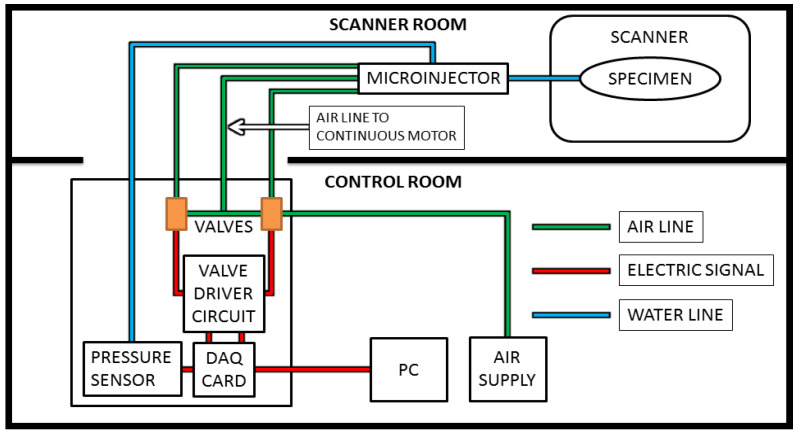
The microinjector is placed near or inside the bore of the magnetic resonance imaging (MRI) scanner. The control box, PC, and air compressor are in the control room, connected to the system as shown.

For this study, the computer is placed in the control room along with the control box, which contains the DAQ card, valves, valve driver circuit, and up to 5psi 2% accuracy pressure sensor (SSCDANT005PGAA5, Honeywell, Golden Valley, MN, USA). The microinjector is attached to a pole located just outside the bore of the MRI, and pneumatic tubes about 3 m long were inserted through the waveguide. The length of the tubing is short enough to cause hysteresis to be negligible, especially given the small amounts of liquid injected for each step and the relatively slow speed. Steps were taken to ensure that no air bubbles were present, and none appeared during the testing as the pressures were low and the tubing was a closed system. In addition, the pressure sensor allowed for control based on feedback, which could allow for longer tubing if needed. The injection line itself measured at approximately 1 m and the specimen was located at the center of the bore. The pressurized air is supplied by an air compressor located in the control room, as illustrated by Figure 2, which illustrates the overall layout schematic for the system.

## 3. Results

### 3.1. Scan Interpretations

**Figure 3:**
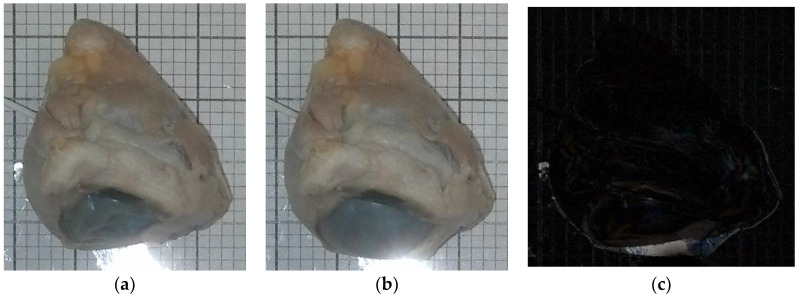
(**a**) (t = 0 s) (pressure = 0 mmHg) (volume injected = 0 mL), (**b**) (t = 535 s) (pressure = 100 mmHg) (volume injected = 1.75 mL), (**c**) absolute difference of (a,b). Light region encompassing eye indicates inflation.

Figure 3a,b are images taken from one of the experiments using the camera and a 10 mL syringe. One specimen was used for testing under MRI. Although some changes in the eyeball’s structure are readily apparent, subtle changes in size were identified by taking the images’ absolute difference using MATLAB’s basic image processing toolbox (Figure 3c). Figure 3a (t = 0 s) had 0 mmHg, and Figure 3b had 100 mmHg after 535 s of continuous water injection (1.75 mL total).

**Figure 4:**
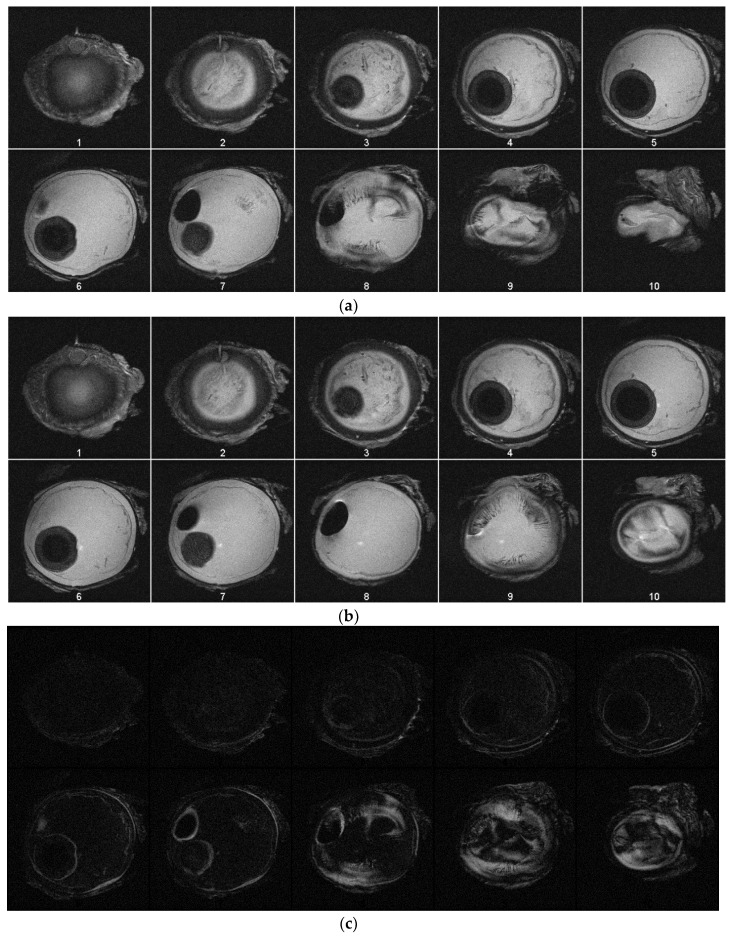
(**a**) Image slices of the specimen at pressure of 0 mmHg. (**b**) Image slices of the specimen at pressure of 100 mmHg. (**c**) Depicted are the processed absolute differences of the images in (a,b), with the lighter regions indicating volumetric expansion due to increased pressure.

**Figure 5:**
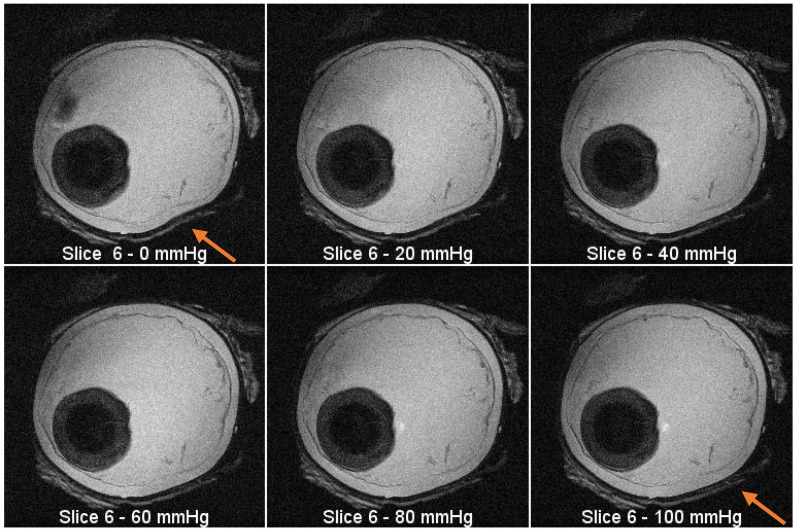
MRI images from the same slice (plane) at increasing pressure intervals. There is visible expansion in the outline of the eyeball as the pressure increases (see arrow location).

MRI images were taken with an actively shielded small animal research Varian Magnex 7 Tesla MRI system with a 72 mm diameter bore and surface coil using the fast spin-echo (FSE) sequence. Images were taken every 20 mmHg starting at 0 mmHg and ending at 100 mmHg, where visible increases in the area of the 2D slice are present. Each image slice is 4 mm thick. Figure 4a, b show the images acquired during MRI tests. Figure 4a contains image slices of the eyeball at different heights while the pressure was 0 mmHg. Figure 4b contains images taken with the eyeball inflated to 100 mmHg. Injection was not continuous for these tests because each scan takes approximately five minutes to complete. For Figure 4a,b, the first image slice is the bottom of the eyeball, and the last image slice is the top of the eyeball. Each image slice is about 4 mm thick. Each image at each pressure is depicted in Figure 5. The opening of the bore is toward the top of each image. Using MATLAB, the absolute difference of Figure 4a,b was taken to highlight subtle changes in size and structure (Figure 4c).

### 3.2. Compliance to MRI Environment

The signal to noise ratio (SNR) generated by the microinjector was tested under three conditions using the spin-echo (SE) sequence of a Varian Magnex 7 Tesla MRI system. All three test conditions were conducted with a phantom placed inside the bore of the magnet. Each test was run for three slices of the phantom, resulting in nine images in total. The baseline for the experiment was conducted with the device completely off. For the next scan, the electronics were powered on, but the motor sat idle. The third scan was made with the motor in operation

The SNR was calculated with the following equation, established in previous literature [14]:

$$SNR=\frac{I_{center}}{SD_{corner}}$$

where *I_center_* is the mean of pixel intensity at the center of an image slice and *SD_corner_* is the standard deviation of the pixel intensity at the corner of an image slice [15,16]. Values were measured using a 40 × 40 pixel square.

**Figure 6:**
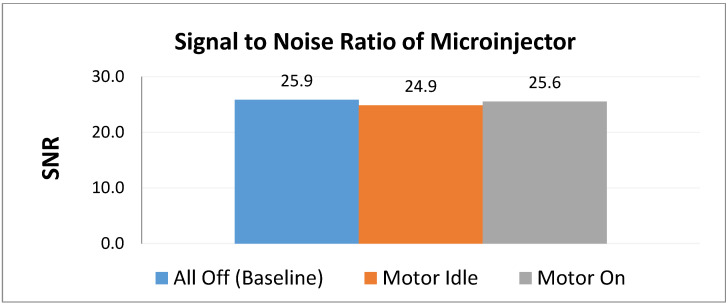
Comparison of relative SNR values of the microinjector under three test conditions.

Figure 6 shows a comparison of the signal to noise ratios generated by each condition. Table 2 shows the comparison of SNR and reduction. On average, the control produced an SNR of around 25.9. With the system powered on and the motor idle, the SNR is approximately 24.9, a reduction of about 3.83%. When the motor is running, the SNR is approximately 25.6, resulting in a reduction of approximately 1.24%. It is expected that when the motor is running that a greater SNR should manifest compared to when the motor is idle. Each condition showed less than 10% deviation from the control SNR, which is determined to be acceptable [14,17].

Table 2 as well as Figure 6 depict the signal to noise ratio and reduction percentages, showing that the signal was not significantly affected.

## 4. Discussion

The results show the feasibility of the system, both in performance and in MRI compatibility. The images taken (Figure 3, Figure 4 and Figure 5) show a visible increase in intraocular pressure as expected, and the conducted image analysis in Figure 4c highlights the changes that occurred within the specimen. The microinjector caused the outer wall of the specimen to expand due to increasing intraocular pressure by injecting liquids, achieving the intended ultimate effect. The system is readily reproducible by 3D printing most parts besides the screw (and would be considered disposable), and relatively inexpensive (less than 500 USD) when compared to commercially existing devices, which are often thousands of USD.

The images showing the absolute difference calculated indicated that there are no perceivable artifacts (undesirable differences) in the images due to the presence of the idle motor or the motor switched on. In addition, the calculated SNR reduction was negligible for all conditions, agreeing with the analysis previously made. Therefore, as predicted, the system is indeed suitable for MRI use.

## 5. Conclusions

The microinjector system with a pressure sensor feedback was successfully able to administer precise volumes of liquid, altering the intraocular pressure of the ex-vivo sample, which is one of the first to have a feedback loop and be MR conditional. It was proven to be suitable for MR use because the SNR reduction was negligible, and the images taken show a clear increase in the volume of the eye specimen. The scope of this project was intended to develop this tool so that it could be used for future research, and the technology behind it may eventually find other applications.

Future research will likely focus on the quantitative analysis of the accuracy and repeatability of the device with various syringe types and sizes. A continuous injection could be monitored by scanning the specimen in rapid succession to create a movie, which could then be analyzed with the same type of image processing software used for the still images. Additional testing could provide valuable insights into the biomechanical structure of the eye and how it responds to increasing pressure over time. Alternative pneumatic actuation (driver) mechanisms with better resolution may be deployed. The control sequence for the device can also likely be further optimized for accuracy by incorporating short bursts or retracting a defined distance after injection to prevent over-pressurizing. At its core, however, the device is intended to explore a new approach to IOP testing and how it relates to glaucoma, and uses a pressure sensor embedded in the line to maintain steady and small injection volumes over a predetermined amount of time. The eventual goal could be to use this or another device or a drug to prevent IOP and reduce the risk of glaucoma-caused blindness in patients around the world.

## Figures and Tables

**Table 1:** Parameters of three different syringe sizes. The maximum number of steps for the 1 mL syringe is rounded down to 2862 steps because half-stepping cannot be achieved.

	Total Volume of Syringe		
	10 mL:	3 mL:	1 mL:
Maximum plunger travel length (mm)	61	42	57.25
Maximum plunger travel length (Steps)	3050	2100	2862
Volume (mL) injected per plunger mm	0.164	0.0714	0.0175
Volume (µL) injected per step	3.28	1.43	0.349

**Table 2:** A comparison of the signal to noise ratio (SNR) reduction using the spin-echo sequence.

	Baseline	Motor Idle	Motor On
SNR	25.9	24.9	25.6
Percent Reduction	0%	3.83% ^1^	1.24% ^1^

^1^ SNR reduction less than 5 percent is typically considered acceptable.
